# Distinctive Combinations of RBD Mutations Contribute to Antibody Evasion in the Case of the SARS-CoV-2 Beta Variant

**DOI:** 10.4014/jmb.2308.08020

**Published:** 2023-11-03

**Authors:** Tae-Hun Kim, Sojung Bae, Sunggeun Goo, Jinjong Myoung

**Affiliations:** Korea Zoonosis Research Institute, Department of Bioactive Material Science and Genetic Engineering Research Institute, Jeonbuk National University, Jeonju 54531, Republic of Korea

**Keywords:** SARS-CoV-2, beta variant, RBD, mutation, antibody

## Abstract

Since its first report in 2019, severe acute respiratory syndrome coronavirus 2 (SARS-CoV-2) has posed a grave threat to public health. Virus-specific countermeasures, such as vaccines and therapeutics, have been developed and have contributed to the control of the viral pandemic, which has become endemic. Nonetheless, new variants continue to emerge and could cause a new pandemic. Consequently, it is important to comprehensively understand viral evolution and the roles of mutations in viral infectivity and transmission. SARS-CoV-2 beta variant encode mutations (D614G, N501Y, E484K, and K417N) in the spike which are frequently found in other variants as well. While their individual role in viral infectivity has been elucidated against various therapeutic antibodies, it still remains unclear whether those mutations may act additively or synergistically when combined. Here, we report that N501Y mutation shows differential effect on two therapeutic antibodies tested. Interestingly, the relative importance of E484K and K417N mutations in antibody evasion varies depending on the antibody type. Collectively, these findings suggest that continuous efforts to develop effective antibody therapeutics and combinatorial treatment with multiple antibodies are more rational and effective forms of treatment.

## Introduction

Severe acute respiratory syndrome virus (SARS-CoV-2), which was first identified in China in late 2019, has posed an unprecedented global health threat. As of March 1, 2023, SARS-CoV-2 infections have resulted in over 680 million cases and 6.8 million deaths. SARS-CoV-2 is a betacoronavirus with a single-stranded, positive-sense RNA genome, which is the largest among known RNA viruses (approximately 32,000 nucleotides). It encodes 16 non-structural proteins (nsp’s), 4 structural proteins, and 9 accessory proteins. The Beta variant of COVID-19, also known as B.1.351, was first identified in South Africa in December 2020 [1]. The World Health Organization (WHO) announced the identification of the variant on December 18, 2020, and classified it as a “variant of concern” on December 24, 2020. Since its emergence, a lot of efforts have been made to develop effective vaccines and therapeutics (reviewed in [2-12] and references therein). Nevertheless, SARS-CoV-2 continues to pose a threat to public health, and the efficacy of antiviral measures need to be improved, particularly against variants, by elucidating the fundamental mechanisms of viral evolution and pathogenesis.

The non-structural proteins are primarily involved in viral RNA synthesis, but research has demonstrated that many of these proteins also play a role in immune evasion through various mechanisms. Coronavirus nsp14 produces an exonuclease that helps to enhance the accuracy of its genome synthesis, reducing replication errors during infection of human coronavirus 229E (hCoV-229E) [13] and SARS-CoV [14]. As a result, coronaviruses generally exhibit a lower mutation rate during replication compared to other RNA viruses such as influenza viruses, roughly by 10-fold [15]. However, due to the high infidelity of the virus-encoded RNA-dependent RNA polymerase (RdRp), coronaviruses can still evolve through mutations, as seen in the case of SARS-CoV-2 [16-18]. Since its emergence in China in late 2019, a series of variants of SARS-CoV-2 have emerged, causing pandemics over the last three years. The beta variant (B.1.352) is one of several variants, first identified in South Africa, which contains four public mutations in the S1 region of the spike protein [19]. Some of these mutations are also found in other variants, including the alpha, gamma, and omicron variants. For example, the E484K mutation.

The beta variant of SARS-CoV-2 contains approximately 10 mutations in its spike protein. Of these mutations, three (K417N, E484K, N501Y) are located in the receptor binding domain (RBD), while the remaining mutations (L18F, D80A, D215G, D242-244, R246I, D614G, A701V) are outside the RBD [20]. The mutations in the RBD are thought to be important for altered ACE2 binding and antibody escape, potentially contributing to increased infectivity and pathogenicity. The D614G mutation, found outside the RBD, has been shown to stabilize the spike protein, resulting in increased viral infectivity [21]. This mutation has been conserved among all SARS-CoV-2 variants since the emergence of the G clade variant, suggesting its critical role in SARS-CoV-2 infections.

In light of these findings, significant scientific efforts have been directed toward determining the roles of individual mutations in SARS-CoV-2 [22-24]. However, it is possible that these mutations may not act alone, but rather have synergistic effects on viral infectivity and pathogenicity. Therefore, we carried out a study to assess the impact of individual mutations and their combinations on viral infectivity and antibody escape. We accomplished this by generating pseudoviruses with wild-type or mutant spike proteins and analyzing the effects of these mutations on viral infectivity and antibody escape. Our findings revealed that the D614G mutation, located outside the RBD region, plays a key role in increasing viral infectivity, while the other mutations have little to no effect. However, when exposed to neutralizing antibodies that target the RBD domain, the RBD mutations were found to contribute to cumulative escape of these antibodies, indicating that they play a more significant role in antibody escape than in infectivity. Collectively, these results suggest that SARS-CoV-2 will continue to evolve by accumulating mutations as a means of evading antiviral immunity posed by natural infections and vaccinations. Our findings may shed light on how to create therapeutic antibodies against SARS-CoV-2 and its variants.

## Materials and Methods

### Cells and Reagents

HEK 293T cells (ATCC, CRL-3216) were grown in Dulbeccós Modified Eagle Medium High Glucose (DMEM; Welgene, Republic of Korea) supplemented with 10% Fetal Bovine Serum (FBS; Welgene, South Korea) and 1%Penicillin-Streptomycin (P/S; Welgene, Republic of Korea). HCC15-ACE2 cells, which are lentiviral transduced HCC15 cells (a lung carcinoma cell line; ATCC, CCL-225), are a stable cell line which expresses cellular receptor of SARS-CoV-2. The stable cells were grown in media supplemented with 10% Fetal Bovine Serum and 1% Penicillin-Streptomycin from Roswell Park Memorial Institute 1640 (RPMI 1640; Welgene, Republic of Korea). HEK 293T and HCC15-ACE2 cells were maintained at 37°C and 5% CO2.

### Mutagenesis of the Spike Protein of the SARS-CoV-2 Wuhan Strain

The spike gene of SARS-CoV-2 was optimized for mammalian expression by the GeneArt (Thermo Fisher Scientific, USA), and cloned into pcDNA3.1 (Invitrogen). To generate Spike proteins with one or more mutations, inverse sequence and ligation independent cloning (SLIC) was performed ([25-28]) with the primers listed below, with mutated codons highlighted. K417N forward; 5'-CAGGCAACATCGCCGATTACAACTACAAGCTGCCCGAC-3', K417N reverse; 5'-CGGCGATGTTGCCTGTCTGTCCAGGAGCGATCTGC-3', E484K forward; 5'-GGCGTGAAGGGCTTCAACTGCTACTTCCCACTGCAG-3', E484K reverse; 5'-GAAGCCCTTCACGCC GTTACAAGGGGTGCTGCCG-3', N501Y forward; 5'-CCTACCTACGGCGTGGGCTATCAGCCCTATAGAGTGGTG-3', N501Y reverse; 5'-CACGCCGTAGGTAGGCTGAAAGCCGTAGGACTGCAG-3', D614G forward; 5'-TATCAGGGCGTGAACTGTACAGAGGTGCCCGTGG-3', D614G reverse; 5'-GTTCACGCCCTGATACAGCACGGCCACCTGATTG-3'. The expression of the spike protein, harboring indicated mutations, was analyzed by Western blotting using polyclonal anti-spike S2 antibodies (SinoBiological, China).

### Generation of Lentivirus Pseudo-typed with SARS-CoV-2 Spike Protein

Pseudo-typed lentiviruses, expressing the SARS-CoV-2 spike protein with or without mutations found in the spike protein of beta variant, were generated by co-transfecting HEK293T cells with plasmids expressing HIV1 gag-pol (psPAX2), SARS-CoV-2 spike (with or without mutations), a lentiviral vector backbone (pHR encoding firefly luciferase) as described before ([12, 29]). The Trans*IT*-Lenti Transfection Reagent (Mirus Bio LLC, USA) was used for DNA transfection. At 72 h post-transfection, culture supernatants containing pseudoviruses were harvested with subsequent removal of debris by centrifugation (3600 RPM for 5min at 4°C). Pseudoviruses were kept at -80°C until use. Pseudoviruses were titrated using quantitative RT-PCR (qRT-PCR). Briefly, pseudoviral RNA was extracted and purified using the QIAamp Viral RNA Mini Kit (Qiagen, Germany), and subsequently subjected to single-step qRT-PCR using the Lenti-X qRT-PCR Titration Kit (Takara, Japan) according to the manufacturer's instructions.

### Measurement of Pseudovirus Infectivity

Pseudoviruses were titrated and normalized to ensure that the same numbers of virus particles were used for infectivity test before infected into HCC15 receptor stable cells, which are highly susceptible to SARS-CoV-2 infection (unpublished data). Pseudovirus-infected cells were lysed at 72 h post-infection for 5 min on ice with chilled Reporter Lysis 5X Buffer (Promega, USA), and then precipitated by centrifugation (at 15,000 RPM for 15 min at 4°C). 25 ul of each lysate was transferred to a new tube, and mixed with 25ul of Luciferase buffer containing luciferase substrate (Luciferase Assay System, Promega).

### SARS-CoV-2 Beta Variant Virus

SARS-CoV-2 beta variant virus (B.1.351) was obtained from the Korea Center for Disease Control (KCDC, #43382) and amplified once in Vero-E6 cells before use at MOI 0.1. Viruses in the culture supernatants were harvested at 60 h post-infection and virus stocks were titrated by tissue culture infectivity dose 50 (TCID50) as previously described ([30, 31]).

### Neutralization Assay

Either pseudoviruses or live SARS-CoV-2 beta variant viruses were mixed with indicated monoclonal antibodies alone or in combination at 37°C for 1 h before infection into Vero E6 cells. Cells were then extensively washed and incubated for 6 h (live SARS-CoV-2 virus) or 72 h (Pseudoviruses) before being subjected to quantitative RT-PCR or luciferase assay, respectively. The percentage of neutralization was calculated using the following formula: (value(untreated)-value(treated)) × 100/value(untreated) (%). Data were plotted using Origin 8.5, and polynomial regression analysis was performed to calculate the inhibitory concentration 50 (IC50).

### Quantitative Real-Time Reverse Transcription Polymerase Chain Reaction (qRT-PCR)

The RNA was extracted using the GENTI advanced Viral RNA Extract Kit an the GENTI 32 Advanced Automatic Extraction Equipment (GeneAll, Republic of Korea). 140 μl of extracted RNA was used in a qRT-PCR assay with the DiaStar OneStep Multiplex qRT-PCR kit (SolGent, Republic of Korea) with the following set of primers and probe employed: forward primer; 5’-GACCCCAAAATCAGCGAAAT-3’, reverse primer; 5’-TCTGGTTACTGCCAGTTGAA-3’, probe; 5’(FAM)-CGCATTACGTTTGGTGGACCCTCA-(TAMRA)3’.

### Statistical Analysis

Statistical analysis was performed using Student’s *t*-test. Statistical significance was defined as a probability value (*p*-value) of lower than 0.05: *, *p* < 0.05; **, *p* < 0.01; ***, *p* < 0.001; ns, *p* ≥ 0.05). Data are presented as the mean ± standard deviation (SD).

## Results

### The D614G Mutation, Found Outside the Receptor Binding Domain, Seems to play a Determining Role in Increased Infectivity of the SARS-CoV-2 Beta Variant

**Fig. 1:**
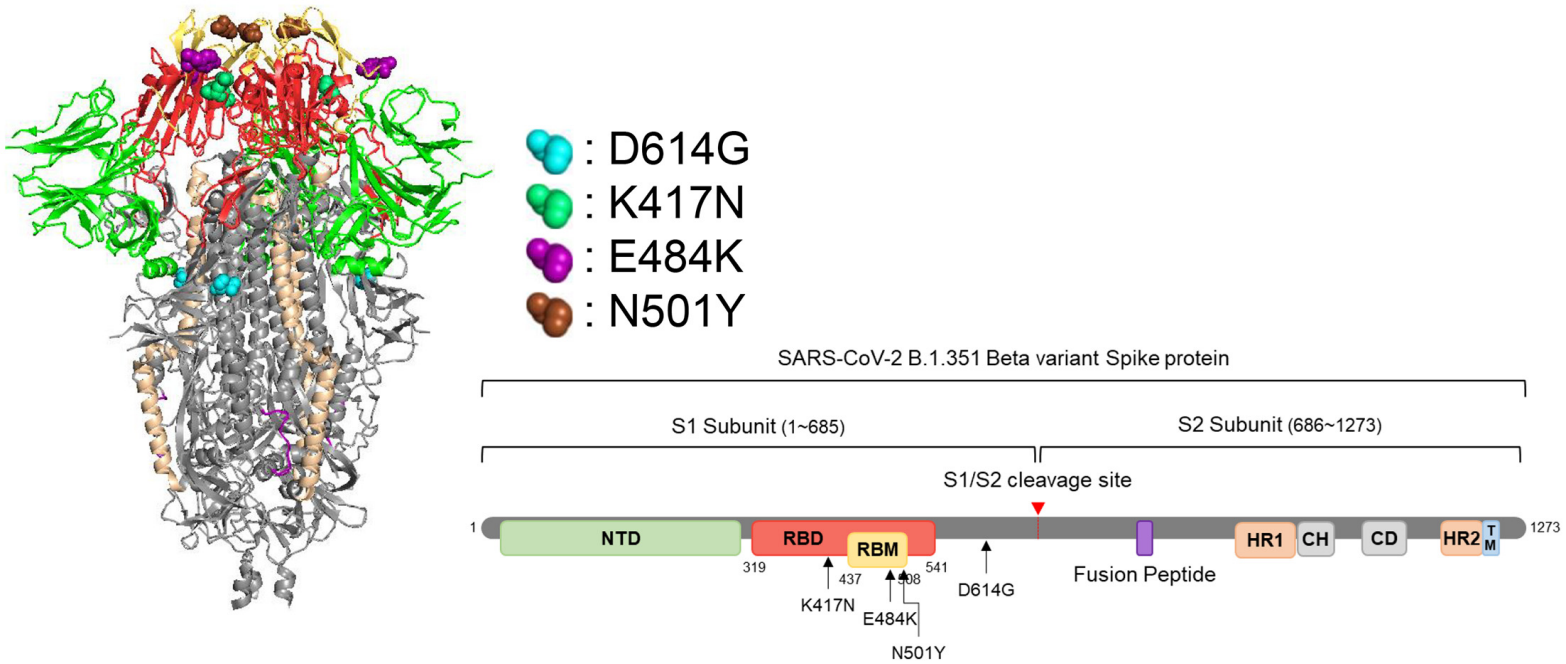
Predicted structure of the spike protein of beta variant. Structural coordinates of the Wuhan spike protein were downloaded from the RCSB protein data bank (# 6VXX), and the predicted structure of the spike protein of the beta variant was generated using the PyMol. The locations of the key mutations in the spike protein are indicated.

**Fig. 2:**
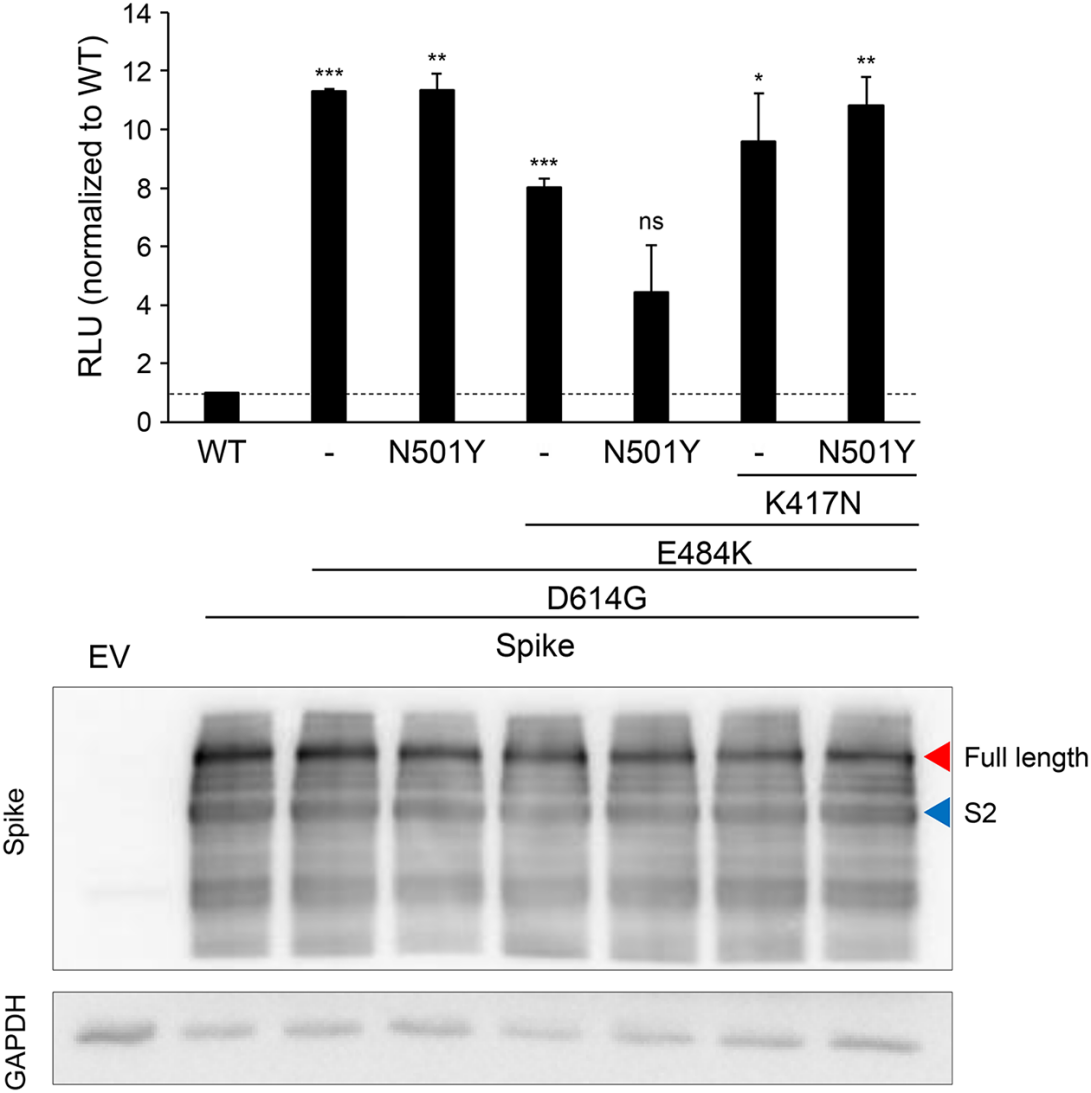
The D614G mutation may have the determining role in the increase of viral infectivity. Pseudoviruses containing one or more mutations in the spike protein were generated as described in the Materials and Methods and infected into Hcc15-ACE2 cells, which stably express human ACE2. At 72 h post-infection, cells were lysed for the luciferase assay. The luciferase activity was normalized to that of the WT spike, and the fold difference in relative luciferase units (RLU) compared to those of WT is plotted. One representative data from three independent experiments is shown (top panel). The expression of the spike protein was analyzed by Western blotting (bottom panel). EV indicates the empty vector transfected control. The size of the full-length spike protein and the S2 cleavage product are depicted on the right. *, *p* < 0.05; **, *p* < 0.02; ***, *p* < 0.001.

The SARS-CoV-2 beta variant contains multiple mutations, including K417N, E484K, N501Y, and D614G. These mutations are located in the spike protein's S1 region, with the first three being in the receptor binding domain (RBD) while D614G is outside of it (Fig. 1). Since the RBD attaches to hACE2 ([32-34]), it is believed that these three mutations in the RBD enhance hACE2 binding while allowing antibodies to escape neutralization [20, 35]. We initially examined if these mutations, either separately or in combination, enhance viral infectivity. Pseudoviruses were created with each of the mutations and were used to infect Hcc15 stable cells (see Materials and Methods) and their comparable spike expression upon infection was evident (Fig. 2, bottom). Interestingly, the pseudovirus containing the D614G mutation alone demonstrated an over 11-fold increase in infectivity compared to the wild-type Wuhan strain, consistent with previous reports ([36, 37]). None of the other mutations, in combination with D614G, showed any further increase in viral infectivity. Moreover, in the presence of the combination of mutations (D614G, N501Y, and E484K), the enhancement of viral infectivity conferred by D614G was nullified, suggesting for an existence of a structural limitation. Interestingly, the pseudoviruses that had D614G/N501Y and D614G/E484K mutations displayed somewhat reduced infectivity, indicating that these combinations might have some negative effects on the virus's structure (Fig. 2, top middle bars). Nevertheless, the presence of K417N could counteract these effects (Fig. 2, top right bars), suggesting that each RBD mutation plays a role in maintaining the proper structure for binding with hACE2 receptors and complements the structural fitness of other mutations to achieve this.

### K417N and E484K Demonstrated a Synergistic Antagonism against Casirivimab-Mediated Neutralization

**Fig. 3:**
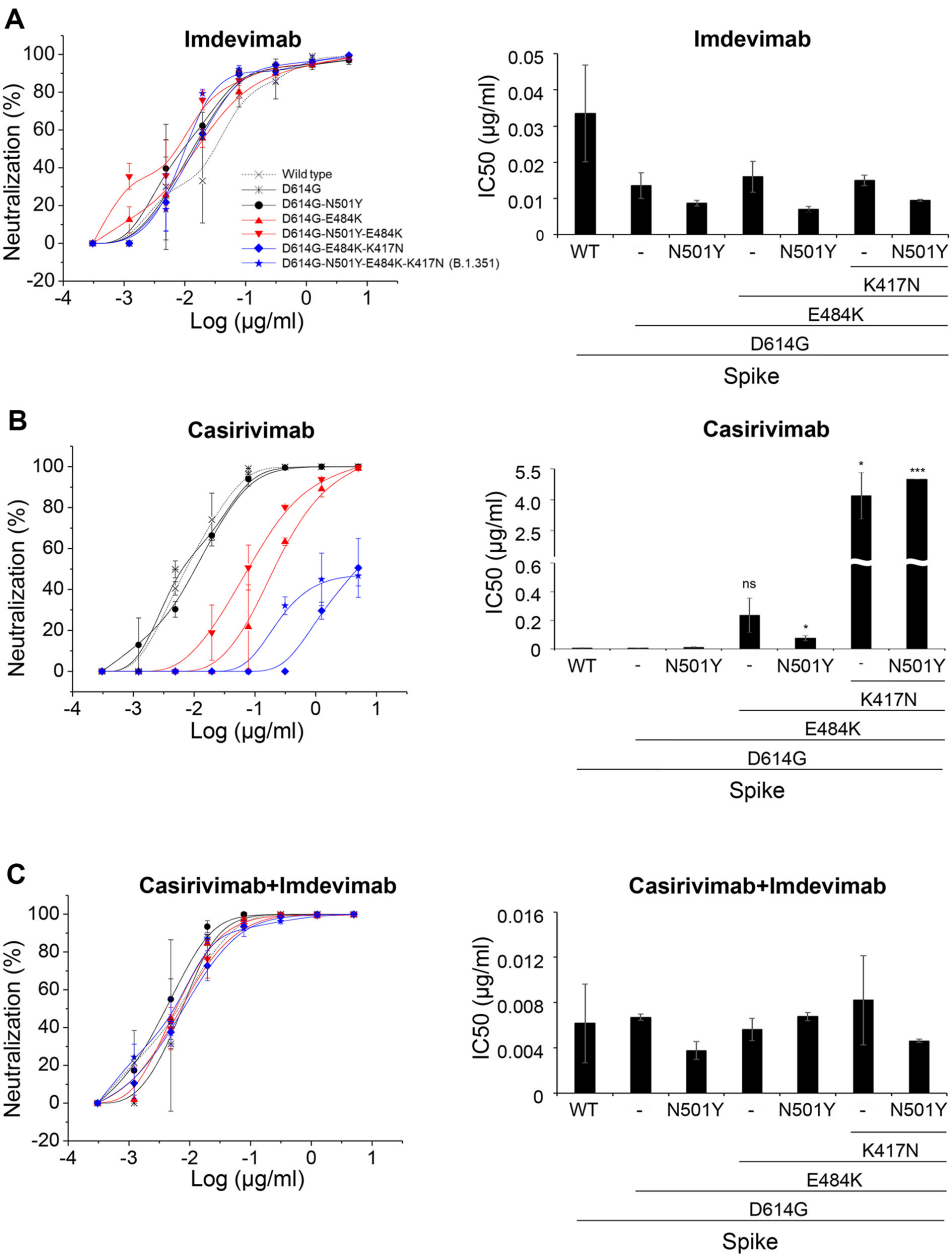
Casirivimab displayed severely impaired neutralizing activity against the spike protein when K417N mutation is introduced. Casirivimab or Imdevimab was, either alone (**A** and **B**) or in combination (**C**), mixed with pseudoviruses bearing various mutations and incubated at 37°C for 1 h before wash. Cells were incubated for 72 h prior to being subjected to luciferase activity. Neutralization percentage, compared to antibody-untreated pseudovirus alone, was calculated and plotted (left panels) and antibody’s neutralization IC50 was calculated as described in the Materials and Methods and IC50’s from two independent experiments were averaged and plotted (right panels). *, *p* < 0.05; ***, *p* < 0.001.

Fig. 2 shows that none of the RBD mutations contribute to the enhancement of viral infectivity. Therefore, it is possible that these mutations may be involved in immune escape mechanisms. Previous studies have demonstrated that mutations such as K417, E484K, and N501Y may play a role in antibody escape. However, these roles have been analyzed individually, and it is not known whether there is an additive or synergistic effect when these mutations are combined. To investigate this, pseudoviruses were generated with individual mutations or combinations of mutations (as shown in Fig. 2), and Regeneron antibodies (Imdevimab or Casirivimab), which were one of the first therapeutic antibodies authorized that target the RBD domain, were used to determine the degree of antibody escape conferred by these mutations (as shown in Fig. 3). It was observed that neutralization by Casirivimab was significantly impaired in the presence of the E484K mutation, while that by Imdevimab was not affected. The combination of Casirivimab and Imdevimab at a 1:1 ratio was able to effectively neutralize pseudovirus infectivity, likely due to the potent neutralization by Imdevimab. It is worth noting that pseudoviruses carrying E484K mutations alone or in combination with N501Y and/or K417N (as shown in Fig. 2A, right panel) displayed increasing levels of resistance to neutralization by Casirivimab, indicating that E484K mutation is a critical factor in viral escape from this antibody. The potency of neutralization decreased depending on the nature and number of mutations, with the order being: D614G (IC50=0.012) < D614G/E484K (IC50=0.097) < D614G/E484K/K417N (IC50=1.187).

**Fig. 4:**
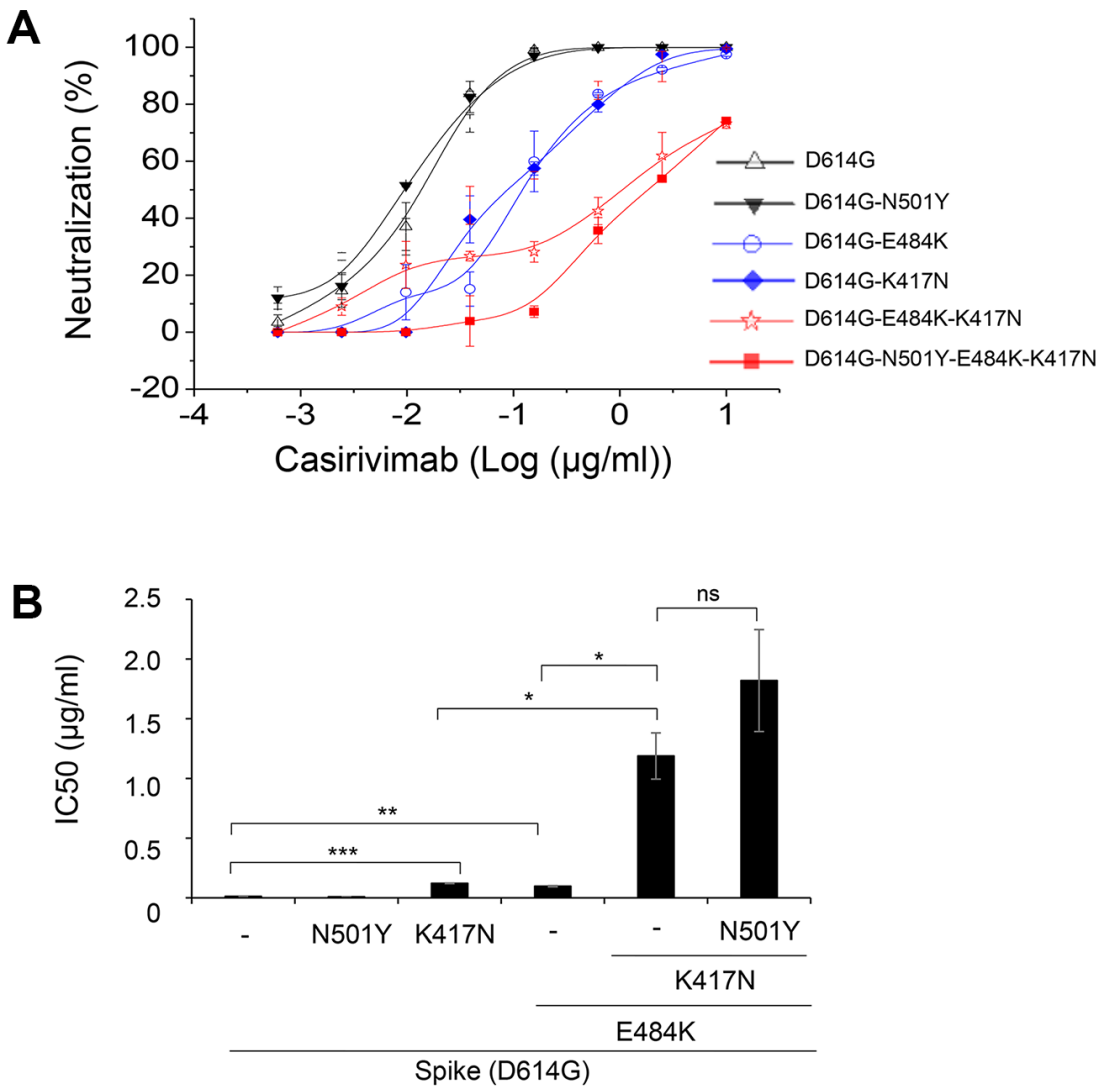
E484K and K417N demonstrated a synergistic effect on antibody escape. Pseudoviruses, which express spike proteins harboring the following mutations: D614G or D614G-N501Y (black lines), D614G-E84K or D614G-K417N (blue lines), D614G-E484K-K417N or D614G-N501Y-E484K-K417N (red lines), were mixed with Casirivimab at 37°C for 1 h before infection into Hcc15-hACE2. At 72 h post-transfection, luciferase activities were measured and normalized to those of pseudoviruses that express spike proteins harboring D614G mutation. One representative data from two independent experiments is shown (top panel). IC50 was calculated by polynomial regression as described in the Materials and Methods and plotted (bottom panel). *, *p* < 0.05; **, *p* < 0.02; ***, *p* < 0.001.

Pseudoviruses that carried both E484K and K417N exhibited a 99-fold increase in the IC50 value compared to those that carried E484K alone (with a 12-fold increase) or none of the mutations (as seen in the right panel of Fig. 3B). This suggests that K417N may have a synergistic effect on the immune escape caused by E484K. To test this hypothesis, we created pseudoviruses that only carried K417N (as shown in Fig. 4) and compared the neutralization rates exhibited by the indicated pseudoviruses, with IC50 values calculated using polynomial regression. In comparison to D614G alone (with an IC50 of 0.012) or D641G/N501Y (with an IC_50_ of 0.009), D614G/E484K (with an IC50 of 0.12) or D614G/K417N (with an IC_50_ of 0.1) showed significantly reduced neutralization rates, with IC50 values that were 10- or 9-fold higher. This indicates that the introduction of either E484K or K417N mutation alone in the spike protein considerably impairs the virus neutralization by Casirivimab. Furthermore, the combination of both E484K and K417N mutations resulted in a 98-fold increase in the IC_50_ value, indicating a potent synergy between the two mutations in enabling the escape of the virus from Casirivimab.

### Casirivimab Displayed Severely Impaired Neutralization Activity against the Beta Variant Virus of SARS-CoV-2

**Fig. 5:**
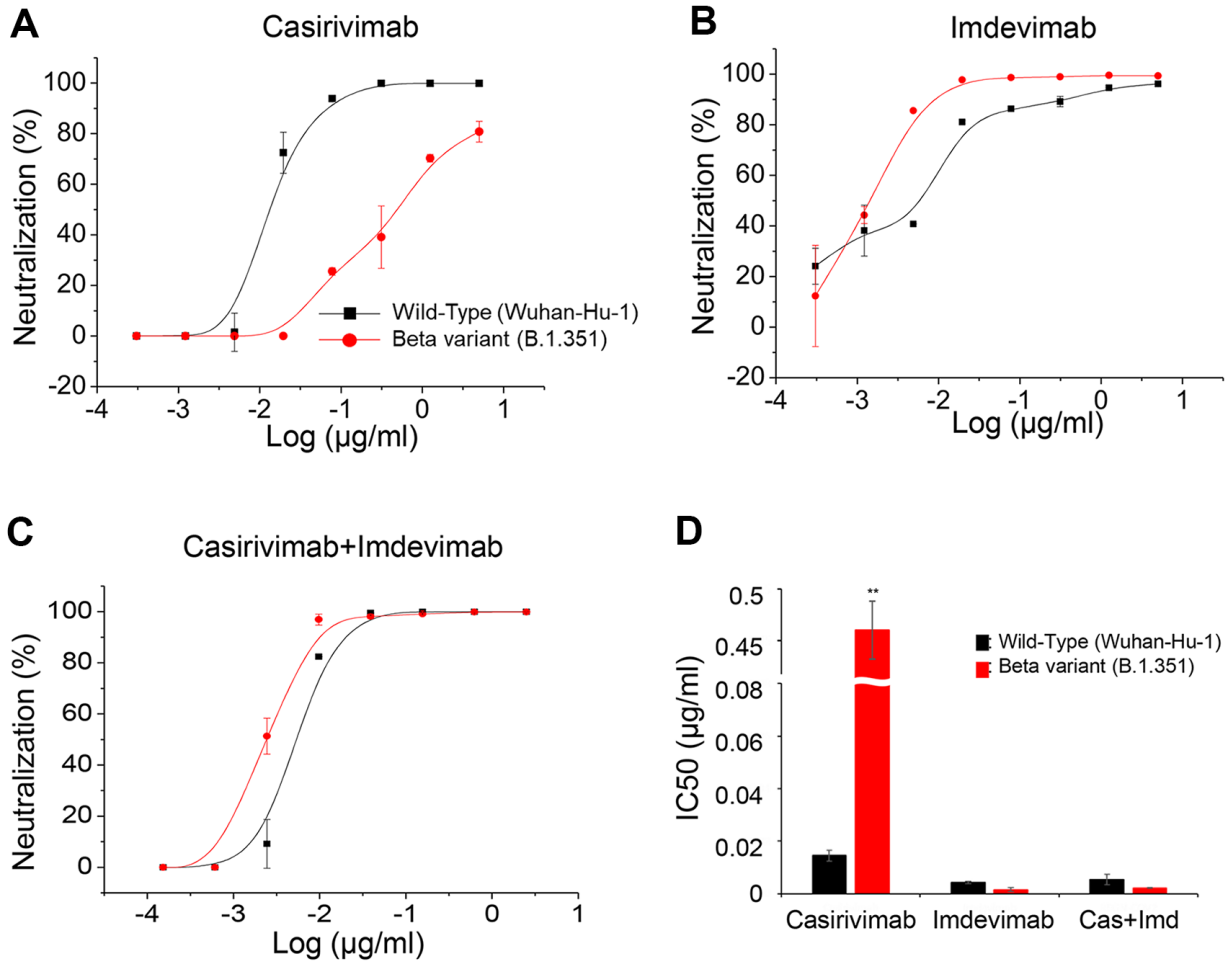
Casirivimab demonstrated severely crippled neutralizing activity against the SARS-CoV-2 beta variant virus. The SARS-CoV-2 Wuhan or beta variant virus was mixed with Casirivimab or Imdevimab, either alone (**A** and **B**) or in combination (**C**) and incubated at 37°C for 1 h before washed three times. Cells were further incubated for 6 h. Viral RNA in the cells was extracted and viral genome equivalents were determined by qRT-PCR as described in the Materials and Methods, and normalized to those of the antibody-untreated control. Neutralization percentage was calculated and plotted (**A**-**C**). IC50 of the monoclonal antibody is shown in D. One representative data from two independent experiments is shown. **, *p* < 0.02.

The pseudoviruses study suggested that the presence of E484K and K417N mutations could significantly reduce the neutralization of SARS-CoV-2 beta variant viruses by Casirivimab, but not by Imdevimab (as shown in Fig. 5). This was confirmed by the fact that the neutralization of beta variant viruses was only affected when Casirivimab was used, not Imdevimab or a combination of both (Fig. 5D). The IC50 value for neutralization of beta variant viruses by Casirivimab (0.46) was roughly 90-fold higher than that of the Wuhan strain (0.01), indicating the crucial role of these two mutations in enabling the virus to escape from the antibody.

### E484K, K417N, and N501Y Acts Synergistically to Evade Regdanvimab-Mediated Inhibition of ACE2-Spike Interactions

**Fig. 6:**
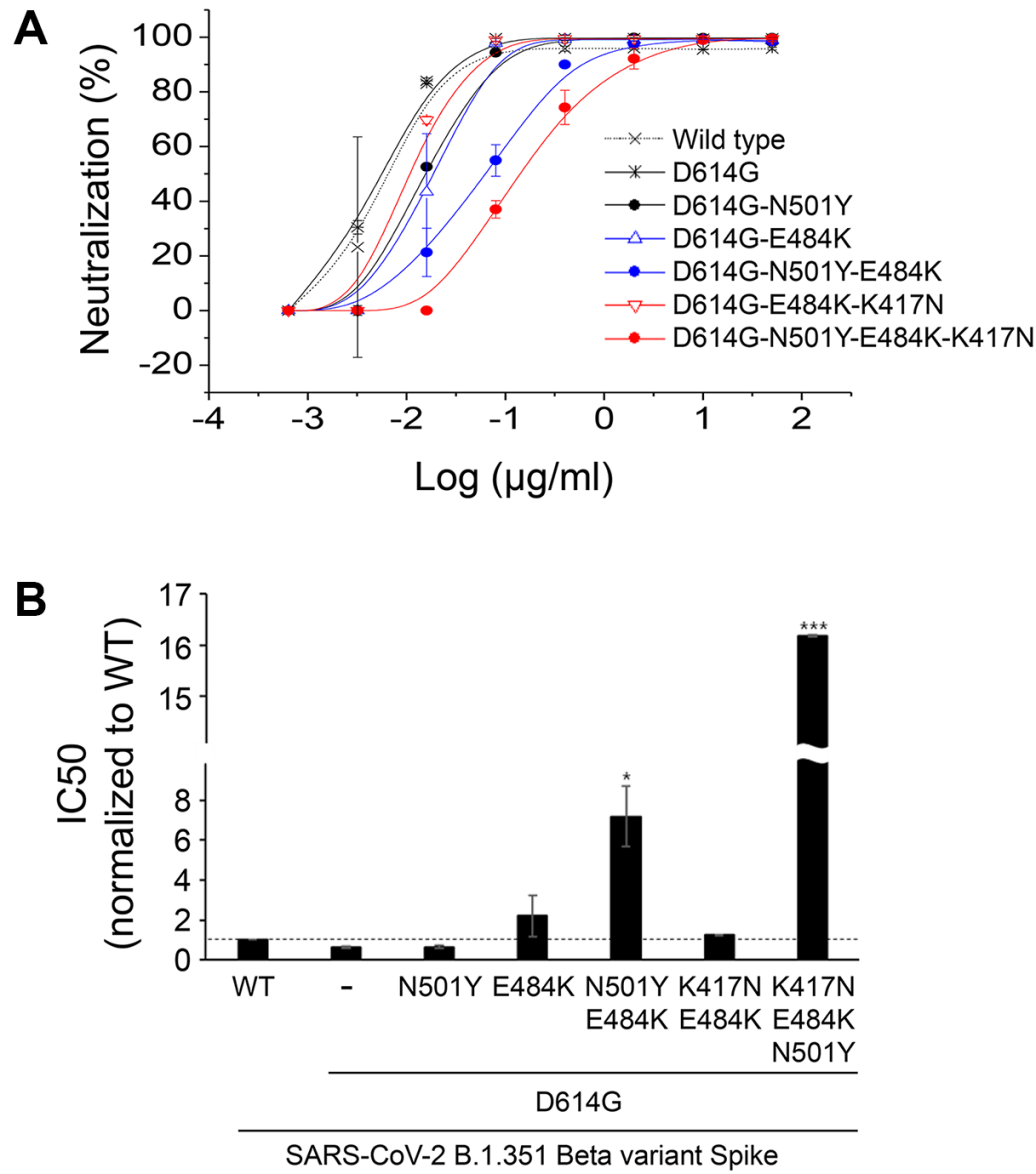
N501Y, E484K, and K417N mutations display a synergistic effect on neutralization by a monoclonal antibody. Pseudoviruses that express spike proteins harboring indicated mutation(s) were mixed with Regdanvimab at 37°C for 1 h before infection into Vero-E6 cells. At 72 h post-infection, cells were lyzed for luciferase activities which were measured and normalized to those of the WT spike protein (**A**). IC50 values of the monoclonal antibody were calculated and plotted in **B**.

In the case of Casirivimab treatment (Fig. 3), N501Y has minimal, if any, inhibitory effects on viral infection, whereas E484K and K417N appear to act in concert against pseudoviral infectivity. To investigate whether this is also true to other therapeutic antibodies, Regdanvimab was employed (Fig. 6) and tested for its inhibitory efficacy against pseudoviral infection with or without a series of mutations described above. Interestingly, N501Y and K417N play a synergistic role against viral infection, whereas E484K have a minimal effect, suggesting that spike-encoded mutations act alone or in different combinations depending on the type antibody used.

## Discussion

Mutations in the spike protein of the SARS-CoV-2 beta variant, also called B.1.351 or the South African variant, have been of particular concern. Some of those mutations, including E484K, K417N, and N501Y, reside in the receptor-binding domain (RBD) of the spike protein, which interacts with the human ACE2 receptor to gain entry into cells [38]. The potential impact of these mutations on infectivity is a major concern. According to research, the beta variant of SARS-CoV-2 is more transmissible than earlier strains, possibly due to its ability to bind more tightly to the human ACE2 receptor [39, 40]. The D614G mutation, found outside the RBD, in SARS-spike CoV-2's protein, which appeared early in the pandemic, has been linked to increased viral infectivity and higher viral loads in infected individuals [6]. The beta variant of SARS-CoV-2 also carries this mutation, which stabilizes the spike protein trimers and thus provides a transmission advantage [7]. In addition, the N501Y mutation in the spike protein of the SARS-CoV-2 beta variant has been associated with increased viral infectivity and transmission by enhancing the binding affinity of the spike protein to the human ACE2 receptor [41, 42]. This increased affinity may explain the higher viral loads observed in individuals infected with the beta variant, as well as its greater transmissibility and vaccine efficacy compared to earlier strains of SARS-CoV-2 [43], highlighting the importance of continued surveillance and research on the evolution and spread of the SARS-CoV-2 virus. Further study is required to fully comprehend the roles of both mutations in SARS-CoV-2 infectivity.

Here, we demonstrated that mutations encoded in the spike protein of SARS-CoV-2 beta variant act synergistically in different combinations, depending on the tested antibodies. When Casirivimab was used, E484K and K417N mutations play a role in antibody escape, either singly or in synergy, whereas N501Y had a minimal effect (Figs. 3-5). In contrast, N501Y and K417N act synergistically in Regdanvimab (Fig. 6). In consistent with pseudoviral studies, Casirivimab exhibited a significantly attenuated neutralizing activity against SARS-CoV-2 beta variant virus. These findings strongly suggest that efforts must be made to develop effective therapeutic antibodies against the ever-changing SARS-CoV-2 which will appear in the future.
